# An [Fe^III^_34_] Molecular Metal Oxide

**DOI:** 10.1002/anie.201911003

**Published:** 2019-10-11

**Authors:** Alice E. Dearle, Daniel J. Cutler, Hector W. L. Fraser, Sergio Sanz, Edward Lee, Sourav Dey, Ismael F. Diaz‐Ortega, Gary S. Nichol, Hiroyuki Nojiri, Marco Evangelisti, Gopalan Rajaraman, Jürgen Schnack, Leroy Cronin, Euan K. Brechin

**Affiliations:** ^1^ EaStCHEM School of Chemistry The University of Edinburgh David Brewster Road Edinburgh EH93FJ UK; ^2^ WestCHEM School of Chemistry The University of Glasgow University Avenue Glasgow G12 8QQ UK; ^3^ Department of Chemistry Indian Institute of Technology Bombay Mumbai 400076 India; ^4^ IMR Tohoku Univ Katahira 2-1-1, Aobaku Sendai 980-8577 Japan; ^5^ Instituto de Ciencia de Materiales de Aragón CSIC-Universidad de Zaragoza 50009 Zaragoza Spain; ^6^ Fakultät für Physik Universitat Bielefeld Postfach 100131 33501 Bielefeld Germany

**Keywords:** DFT calculations, Fe^III^ cages, magnetic behaviour, molecular metal oxides, spin frustration

## Abstract

The dissolution of anhydrous iron bromide in a mixture of pyridine and acetonitrile, in the presence of an organic amine, results in the formation of an [Fe_34_] metal oxide molecule, structurally characterised by alternate layers of tetrahedral and octahedral Fe^III^ ions connected by oxide and hydroxide ions. The outer shell of the complex is capped by a combination of pyridine molecules and bromide ions. Magnetic data, measured at temperatures as low as 0.4 K and fields up to 35 T, reveal competing antiferromagnetic exchange interactions; DFT calculations showing that the magnitudes of the coupling constants are highly dependent on both the Fe‐O‐Fe angles and Fe−O distances. The simplicity of the synthetic methodology, and the structural similarity between [Fe_34_], bulk iron oxides, previous Fe^III^–oxo cages, and polyoxometalates (POMs), hints that much larger molecular Fe^III^ oxides can be made.

## Graphical abstract

**Fe Maiden**: An [Fe_34_] molecular metal oxide consists of an [Fe_4_] tetrahedron encapsulated in a [Fe_18_] truncated tetrahedron, face‐capped by four [Fe_3_] triangles, and is characterised by alternate layers of tetrahedral and octahedral Fe ions linked by oxide and hydroxide anions.

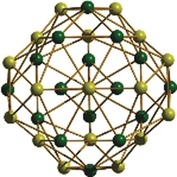

It is interesting to note the enormous size difference between the largest polyoxometalates (POMs), most commonly constructed from high oxidation state, diamagnetic metal ions,^1^ and molecules built from the high spin (d^5^), paramagnetic Fe^III^ ion,^2^ despite both often containing similar metal oxide cores.^3^ The most pertinent examples of Fe^III^ clusters conforming to POM‐like architectures are [Fe_13_]^4^ and [Fe_17_];^5^ both are structurally related with alternating layers/shells of tetrahedral and octahedral metal ions—the former has the α‐Keggin structure,4a and the latter the ϵ‐Keggin structure with four additional capping metal ions.^5^ In addition, the much studied [Fe^III^_30_] icosidodecahedron,^6^ demonstrates that very large (heterometallic) molecular metal oxides containing paramagnetic metal ions can (a) be synthesized, (b) retain POM‐like architectures, and (c) possess fascinating physical properties—the high symmetry icosidodecahedron possessing geometric spin frustration.^7^ This has prompted us to speculate that large and very large Fe^III^ molecular metal oxides, approaching the size and structural diversity of POMs, can be constructed, but with the terminal oxides replaced by simple monodentate ligands. There appears to be no chemical reason why such species cannot form, and their synthesis would help bridge the “gap” between the fields of molecular magnetism (where the vast majority of molecules have nuclearities less than twenty) and POM chemistry (where complexes can be an order of magnitude larger), producing species with a myriad of potentially interesting physical properties, with applications in chemistry, physics, materials science, biology and medicine.^8^

The [Fe_17_] complex in particular hints at a potentially successful route to the synthesis of such species. It is made very simply by dissolving anhydrous FeX_3_ (X=Cl, Br) in wet pyridine (or any analogous liquid base such as β‐picoline, iso‐quinoline, ethyl‐pyridine, lutidine, etc).^9^ The wet pyridine appears to fulfill at least five simultaneous roles: it is the solvent, the base, the source of water (hence oxide), monodentate ligand (with the halide ions) that encases the metal oxide core, and source of the charge balancing pyridinium cations. Interestingly, in POM chemistry the addition of (bulky) organic amine cations is thought to aid the self‐asembly of large nuclearity species by isolating the smaller building blocks, preventing rapid aggregation into complexes with (smaller) stable spherical topologies.^10^ Herein we discuss the synthesis, structure and magnetic behaviour of [Fe^III^_34_(μ_4_‐O)_4_(μ_3_‐O)_34_(μ_2_‐OH)_12_Br_12_(py)_18_]Br_2_ (**1**) (Figure 1, S1–S5 in the Supporting Information) which is made via just such a strategy, through a small modification (the addition of either hexamethylene tetramine (HMTA) or morpholine) in the preparation of [Fe_17_].^11^

**Figure 1:**
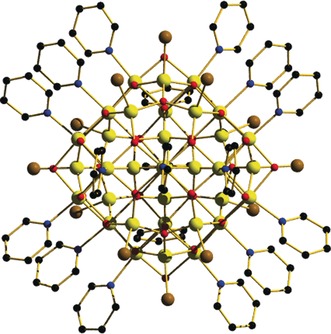
Molecular structure of the cation of **1**. Colour code: Fe=yellow, O=red, N=blue, C=black, Br=brown. H atoms and anions omitted for clarity.

Compound **1**, crystallises in the monoclinic space group *P*2_1_/*n* with the full complex in the asymmetric unit. The metallic skeleton (Figure 2, S2, S3) describes a [Fe^III^_4_] tetrahedron encapsulated within a [Fe^III^_18_] truncated tetrahedron, whose large faces are capped by [Fe^III^_3_] triangles. The (sixteen) metal ions in the inner tetrahedron and the outer triangles are all tetrahedral and the (eighteen) Fe ions in the truncated tetrahedron are all octahedral. The presence of tetrahedral‐octahedral–tetrahedral “shells” of metal ions is as found in the [Fe_13_] and [Fe_17_] complexes (Figure 3) and Fe containing minerals, such as magnetite and maghemite. The inner tetrahedron is connected to the [Fe^III^_18_] truncated tetrahedron via ten (4×μ_4_; 6×μ_3_) O^2−^ ions (Figure S2). Each face‐capping, oxo‐centred [Fe_3_] triangle is connected to the [Fe^III^_18_] truncated tetrahedron via six μ_3_‐O^2−^ ions. The remaining twelve μ_2_‐OH^−^ ions link the metal ions situated on the triangular faces on the truncated tetrahedron. Each of the octahedral metal ions in the [Fe_18_] truncated tetrahedron and tetrahedral metal ions in the face capping [Fe_3_] triangles have their coordination geometries completed through the presence of a pyridine (py) molecule and a bromide, respectively. The Br counter anions are associated with the triangular faces of the truncated tetrahedron with Br⋅⋅⋅(μ_2_‐)O distances in the range 3.21–3.48 Å, and the py C‐atoms on a neighbouring molecule (Br⋅⋅⋅C, 3.46 Å). The other prevalent intermolecular interactions occur between adjacent Br ions and py molecules (Br⋅⋅⋅C, 3.50 Å).

**Figure 2:**
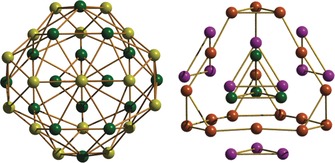
Alternative views of the metallic skeleton of **1**. Left: highlighting the layers of tetrahedral (green) and octahedral (yellow) metal ions. Right: highlighting the central [Fe_4_] tetrahedron (green) encapsulated by the [Fe_18_] truncated tetrahedron (brown) whose large faces are capped by [Fe_3_] triangles (pink).

**Figure 3:**
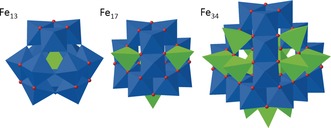
Polyhedral representation comparing the structures of [Fe_13_] (left),4a [Fe_17_] (centre)^5^ and [Fe_34_] (right). Tetrahedral Fe^III^=green, octahedral Fe^III^=blue.

Magnetic measurements of **1** strongly hint at rather large, competing, antiferromagnetic exchange interactions between the Fe centres. The susceptibility data (*T=*350–2 K, *B*=0.1 T; Figure 4 a) shows that the *χT* value at *T=*350 K (≈60 cm^3^ K mol^−1^) is well below the Curie constant expected for thirty four uncoupled Fe^III^ ions (150 cm^3^ K mol^−1^). As temperature is decreased the value of *χT* first increases to a broad maximum of ≈75 cm^3^ K mol^−1^ centred around *T=*150 K, before dropping slowly to a value of ≈70 cm^3^ K mol^−1^ at *T=*50 K. Below this temperature the value drops significantly, and is strongly field dependent. Magnetisation (*M*) data (Figure 4 b) show an initial, fairly rapid, increase to a value of ≈12 μ_B_ (for *T=*2 K, *B*<2 T) before first plateauing and then increasing in a more linear fashion to *B*=7 T where *M*≈15 μ_B_. This linear like increase is continued in the *B*=7–35 T field range (Figure 4 c), where *M* reacheas a maximum value of ≈30 μ_B_. The low temperature susceptibility and magnetisaton data suggest a relatively small magnetic moment, in agreement with heat capacity measurements where the zero‐field magnetic entropy content reaches just *S=*1.6 *R* at *T*=2 K (Figure S6).

**Figure 4:**
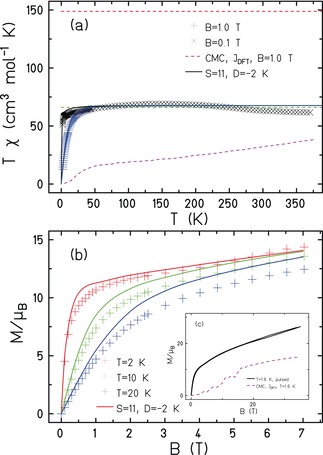
a) Magnetic susceptibility: symbols denote measurements, the red dashed line marks the paramagnetic limit for thirty four *S=*5/2 ions, the green dashed line the respective value for *S=*11. The solid curves belong to single‐spin calculations, the dashed magenta curve to CMC simulations. b) Low‐field magnetisation: the solid curves depict the single‐spin magnetisation. c) Pulsed‐field magnetisation (solid curve) compared to (b) and CMC.

A simple explanation of the temperature and field dependence of the magnetic data is not straightforward. Since the cluster is much too large for a quantum calculation in a spin model such as the Heisenberg model we resort to two approximations. Classical Monte Carlo calculations (CMC) of a classical Heisenberg model, that often deliver accurate results in the temperature and field regions where quantum effects are of minor importance, that is, at elevated temperatures compared to the exchange couplings,7a suggest an even smaller susceptibility and magnetisation compared to the experiment, when using the exchange parameters provided by DFT (see Table 1). In view of the relatively strong exchange, we surmise that we are always in the quantum regime, where the classical calculations point us in the right direction, but are poor approximations otherwise. Guided by the nearly flat high temperature *χT* data, we investigated a quantum model where we assume a low‐lying level structure similar to a zero‐field split total spin *S*=11 with *g* fixed at *g*=2.04, as obtained from HFEPR measurements (Figure S7). This effective model fits both *χT* vs. *T* and *M* vs. *B* (Figures 4 a and b) astonishingly well, and it also explains why specific heat measurements detect very few low‐lying levels (compared to a total of 2.8×10^26^ levels). While the absolute numbers in this effective model should be taken with a pinch of salt, they do hint at the presence of non‐neglibile anisotropy. We also expect that the true low‐energy spectrum contains numerous additional (small) spin states, and in the absence of any out‐of‐phase *χ*′′ signals in the ac susceptibility, that may not be of perfect easy axis character.

In order to estimate the magnitude of the magnetic exchange interactions in **1** we have employed a DFT methodology (B3LYP/TZVP) known to yield excellent numerical estimates of *J* values.^12^, ^13^, ^14^, ^15^ Calculations were performed using the model structures shown in Figures S8–S12 (see the computational details in the Supporting Information for discussion). The symmetric nature of the cage reduces the number of unique exchange interactions to five, describing those between: (i) inner tetrahedral Fe^III^ ions (*J*_1_) connected by μ_3_‐O^2−^ ions; (ii) inner tetrahedral and outer octahedral Fe^III^ ions (*J*_2_) connected by μ_3_/μ_4_‐O^2−^ ions; (iii) outer octahedral Fe^III^ ions (*J*_3_) connected by μ_3_‐O^2−^ ions; (iv) outer tetrahedral Fe^III^ ions (*J*_4_) connected by μ_3_‐O^2−^ ions; and (v) outer tetrahedral and outer octahedral Fe^III^ ions (*J*_5_) connected by μ_3_‐O^2−^ ions (Scheme S1). The calculated *J* values are listed in Table 1. The computed exchange coupling constants are all antiferromagnetic in nature and strongly correlated to the Fe−O distances and Fe‐O‐Fe angles, with larger angles and shorter bonds enhancing the antiferromagnetic part of the exchange, in agreement with the magneto‐structural correlation developed by Weihe and Güdel.^16^

**Table 1:** Calculated *J*_DFT_ values for the five unique exchange interactions in **1**, alongside the average Fe‐O‐Fe angles and Fe−O, Fe⋅⋅⋅Fe distances per interaction.

	Fe‐O‐Fe [°]	Fe−O [Å]	Fe⋅⋅⋅Fe [Å]	*J*_DFT_ [cm^−1^]
*J*_1_	118	1.86	3.19	−24.2
*J*_2_	121	1.95	3.39	−38.4
*J*_3_	95.5	1.97	2.92	−15.7
*J*_4_	119	1.92	3.32	−47.3
*J*_5_	129	1.92	3.47	−68.2

Spin density data are provided in Figures S13–S17 and Tables S2–S6. We have computed the overlap integrals for all *J* pairs (Tables S7–S11), which show a direct correlation between the number of orbital interactions and the magnitude of the antiferromagnetic exchange. For example, for *J*_3_ only two dominant overlaps (d_*xy*_||d_*xy*_ and $d_{x^{2}-y^{2}}$ ||d_*xy*_) are detected leading to the smallest calculated *J* value (−15.7 cm^−1^), whereas there are seven different, large interactions for *J*_5_, resulting in the largest *J* value (−68.2 cm^−1^). Note that in the latter, the d_*xz*_ orbital of the tetrahedral Fe^III^ ion is found to overlap significantly with all the d‐orbitals of the octahedral Fe^III^ ion, with the exception of the d_*xy*_ orbital.

In conclusion, the addition of an organic amine (HMTA, morpholine) to a wet py/MeCN solution of FeBr_3_ produces an [Fe^III^_34_] complex, double the size of the cage produced in wet py/MeCN alone, [Fe^III^_17_]. The molecule, whose structure describes an [Fe_4_] tetrahedron encapsulated in a [Fe_18_] truncated tetrahedron, face‐capped by four [Fe_3_] triangles, is characterised by alternate layers of tetrahedral and octahedral Fe ions linked by oxide and hydroxide anions. Magnetic measurements reveal relatively strong, competing AF exchange interactions between the Fe^III^ ions, with DFT calculations suggesting a direct correlation between the number of orbital interactions and the magnitude of the AF exchange. The simplicity of the synthetic procedure and the structural similary of [Fe_34_] to bulk iron oxides such as magnetite and maghemite (Figure S18), and to much larger POMs of high oxidation state, diamagnetic metal ions such as V, W and Mo, hints that a diverse family of novel Fe^III^ molecular metal oxide structures awaits discovery. This, in turn, suggests an exciting route to the bottom‐up formation of molecular metal oxide “nanoparticles” with a raft of potential applications.

## Conflict of interest

The authors declare no conflict of interest.

## Supporting information

As a service to our authors and readers, this journal provides supporting information supplied by the authors. Such materials are peer reviewed and may be re‐organized for online delivery, but are not copy‐edited or typeset. Technical support issues arising from supporting information (other than missing files) should be addressed to the authors.

Supplementary
