# Supplementary material for: An [FeIII 34] Molecular Metal Oxide
Source: Angew Chem Int Ed Engl. 2019 Oct 11;58(47):16903–6. doi: 10.1002/anie.201911003 (PMC7186828; doi:10.1002/anie.201911003)
Supplement: Supplementary file 1 — Supplementary [file ANIE-58-16903-s001.pdf]

Angewandte Chemie
Eine Zeitschrift der Gesellschaft Deutscher Chemiker

Supporting Information

An [Fe^{III}_{34}] Molecular Metal Oxide

Alice E. Dearle, Daniel J. Cutler, Hector W. L. Fraser, Sergio Sanz, Edward Lee, Sourav Dey, Ismael F. Diaz-Ortega, Gary S. Nichol, Hiroyuki Nojiri, Marco Evangelisti, Gopalan Rajaraman, Jürgen Schnack,* Leroy Cronin, and Euan K. Brechin**

anie_201911003_sm_miscellaneous_information.pdf

Synthesis of $[Fe^{III}_{34}(\mu_4\text{-}O)_4(\mu_3\text{-}O)_{34}(\mu\text{-}OH)_{12}Br_{12}(py)_{18}]Br_2$ (1)

$FeBr_3$ (0.591 g, 2 mmol), hexamethylenetetramine (HMTA; 0.476 g, 3.4 mmol) and pyridine (1 mL, 12.3 mmol) were dissolved in MeCN (25 mL). The reaction was left for 2h 45mins with continuous stirring at room temperature. The resulting solution was filtered and left to stand overnight, during which time a small amount of precipitate formed. The precipitate was filtered off and the supernatant layered with acetone. After two weeks, this yielded dark brown, rod-shaped crystals suitable for X-ray diffraction. Anal. Calcd (%) for $C_{90}H_{102}Br_{14}Fe_{34}N_{18}O_{50}$: C 20.58, H 1.96, Fe 36.14, N 4.80; found: C 19.98, H 2.01, Fe 35.88, N 4.52. Yield: 15% based on Fe.

Compound **1** can also be prepared by replacing the HMTA in the above reaction with morpholine (4 mmol) or N-methylmorpholine (4 mmol).

X-ray crystallography

Diffraction data for **1** was collected using a Rigaku Oxford Diffraction SuperNova diffractometer with CuK_{α} radiation, and is given in Table S1. An Oxford Cryosystems Cryostream 700+ low temperature device was used to maintain a crystal temperature of 120.0 K. The structure was solved using ShelXT and refined with version ShelXL interfaced through Olex2.[1],[2] All non-hydrogen atoms were refined using anisotropic displacement parameters. H atoms were placed in calculated positions geometrically and refined using the riding model. CCDC: 1900069.

A unit cell check of the crystals was performed prior to each of the following measurements.

Magnetic Susceptibility and Magnetisation (<7 T)

Dc susceptibility and magnetisation data were measured on powdered, polycrystalline samples of **1** in the T = 2-300 K and B = 0-7 T temperature and field ranges on a Quantum Design MPMS XL SQUID magnetometer equipped with a 7 T dc magnet. Diamagnetic corrections were applied to the data using Pascal's constants.

High Field Pulsed Magnetisation (<35 T)

Low-temperature magnetisation data was measured by the use of a conventional inductive probe in pulsed-magnetic fields, where the temperature reached as low as 1.6 K.[3] The maximum field reached was 35 T. Polycrystalline samples with a typical mass of 15 mg were mounted in a capillary tube made of polyimide. The sample, which was not fixed within the sample tube, was aligned along the magnetic field direction. Magnetisation curves were found to be identical after we applied the magnetic field several times due to the saturation of the orientation effect.

Heat Capacity

Heat capacity data were collected in the temperature range 0.3–20 K using a Quantum Design PPMS equipped with a ^{3}He cryostat. The powdered, polycrystalline sample of **1** was pressed into a thin pellet with mass of about 0.5 mg. Apiezon-N grease was used to facilitate the sample thermalization at low temperatures, and its contribution to the heat capacity was subtracted using a phenomenological expression.

High Field, High Frequency EPR (HFEPR)

HFEPR spectra for polycrystalline samples were obtained on the Terahertz ESR Apparatus (TESRA-IMR) installed in the magnetism division of Institute of Material Research, Tohoku University.[4a] A case made of polyethylene was used for packing the sample. The radiation was produced by Gunn oscillators and backward traveling wave oscillators (BWO).

Table S1. Single crystal X-ray data for **1**, alongside the powder XRD pattern.

Compound **1**			
**Formula**	$C_{95}H_{107}Br_{14}Fe_{34}N_{19}O_{50}$	Z'	1
$D_{calc.}$ **/ g cm^{-3}**	1.777	**Wavelength/Å**	1.54184
μ**/mm^{-1}**	22.968	**Radiation type**	CuK_{α}
**Formula Weight**	5332.63	Θ_{min}/°	3.378
**Colour**	dark red	Θ_{max}/°	68.251
**Shape**	block	**Measured Refl.**	297235
**Size/mm^3**	0.45×0.21×0.17	**Independent Refl.**	36505
T**/K**	120.0	**Reflections Used**	23404
**Crystal System**	monoclinic	R_{int}	0.2035
**Space Group**	$P2_1/n$	**Parameters**	1897
a**/Å**	18.4222(5)	**Restraints**	120
b**/Å**	32.8959(10)	**Largest Peak**	2.270
c**/Å**	33.9586(9)	**Deepest Hole**	-1.311
α/°	90	**GooF**	0.995
β/°	104.444(3)	wR_2 **(all data)**	0.2880
γ/°	90	wR_2	0.2598
**V/Å^3**	19928.9(10)	R_1 **(all data)**	0.1309
Z	4	R_1	0.1044

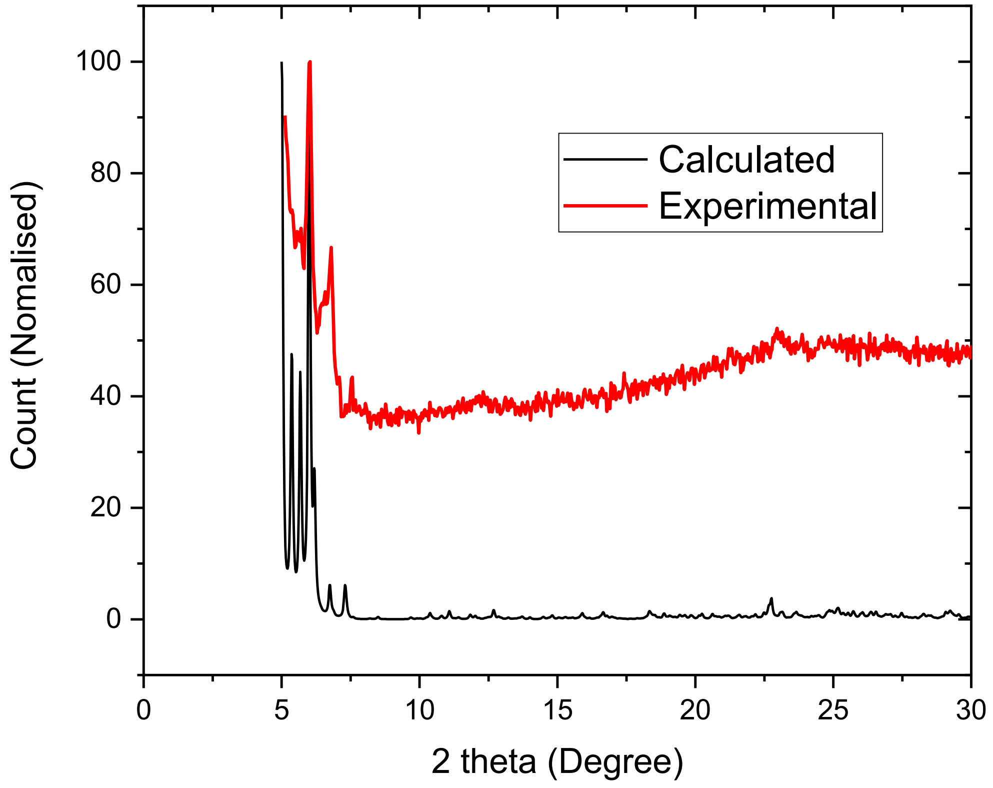

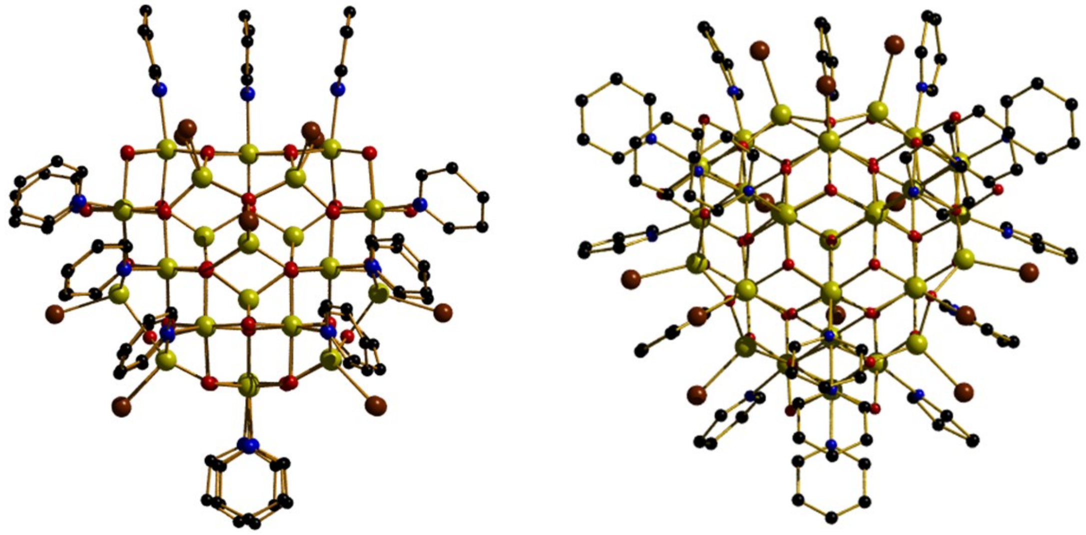

Figure S1. Orthogonal views of the [Fe_{34}] cation. Colour code: Fe = yellow, O = red, C = black, N = blue, Br = brown. H-atoms omitted for clarity.

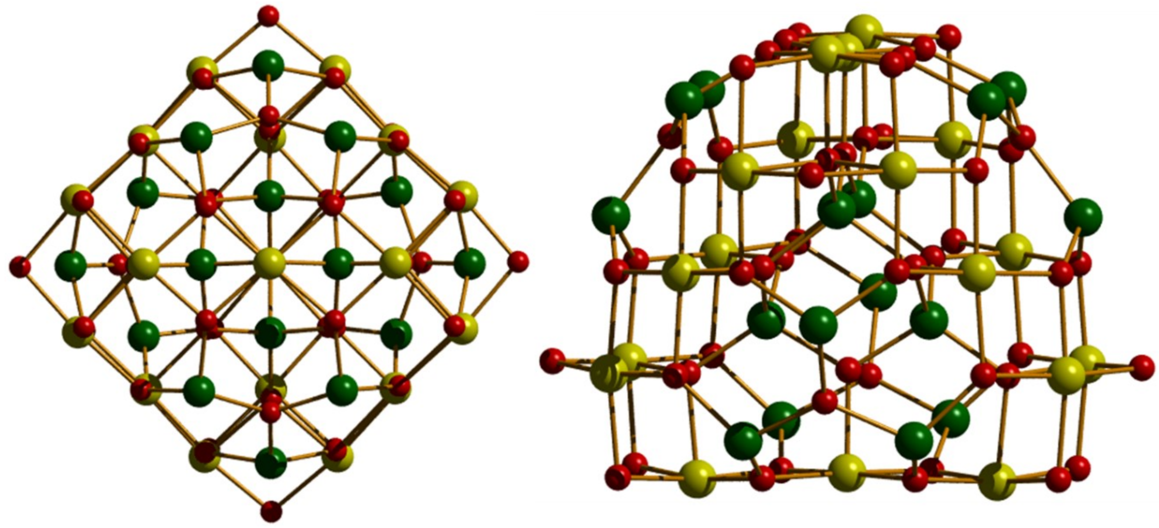

Figure S2. Orthogonal views of the metal-oxygen core of the [Fe_{34}] cation, highlighting the positions of the octahedral (yellow) and tetrahedral (green) Fe ions, and the bridging O (red) atoms.

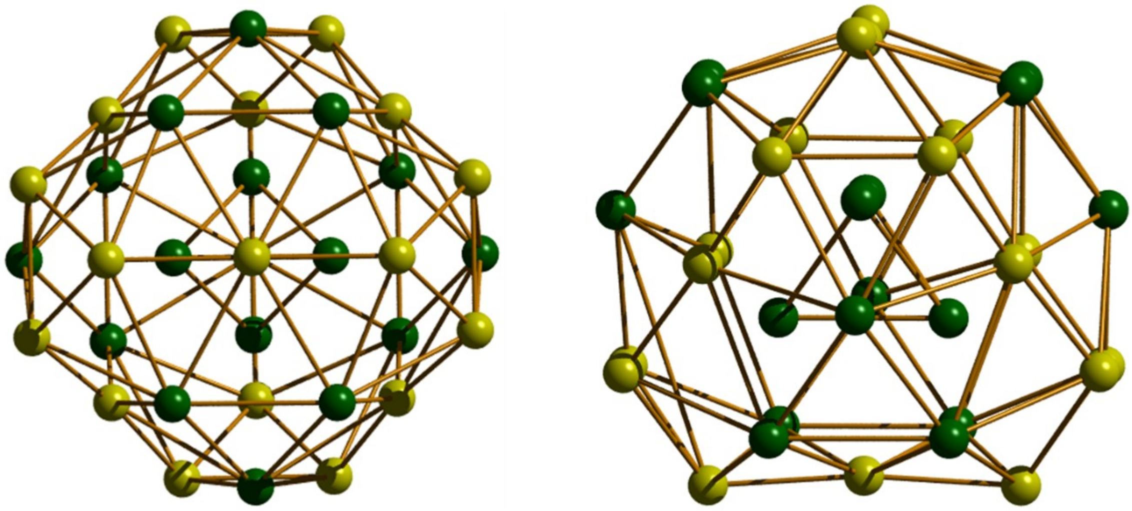

Figure S3. Orthogonal views of the [Fe_{34}] metal polyhedron, highlighting the positions of the octahedral (yellow) and tetrahedral (green) Fe ions.

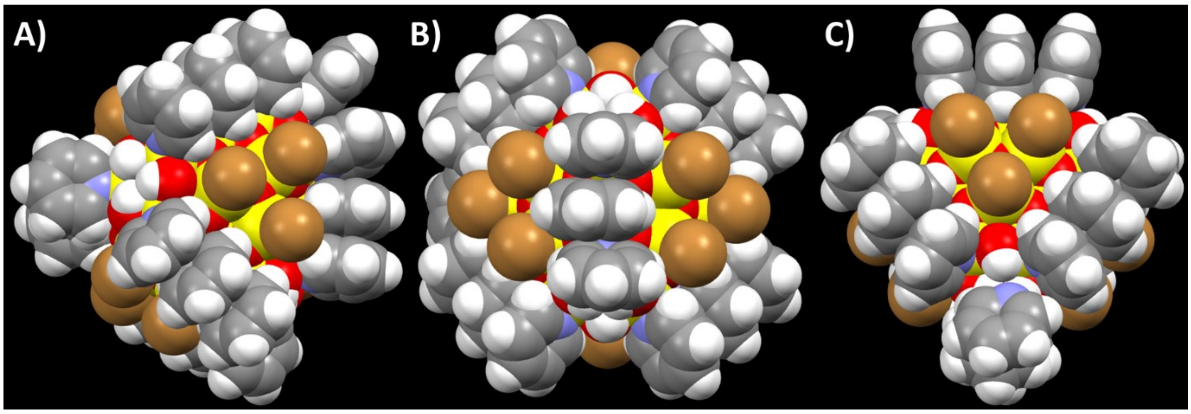

Figure S4. Structure of the [Fe_{34}] cation in **1** in space-fill representation, viewed along the *a*-, *b*- and *c*-axis directions of the unit cell (left-right, respectively).

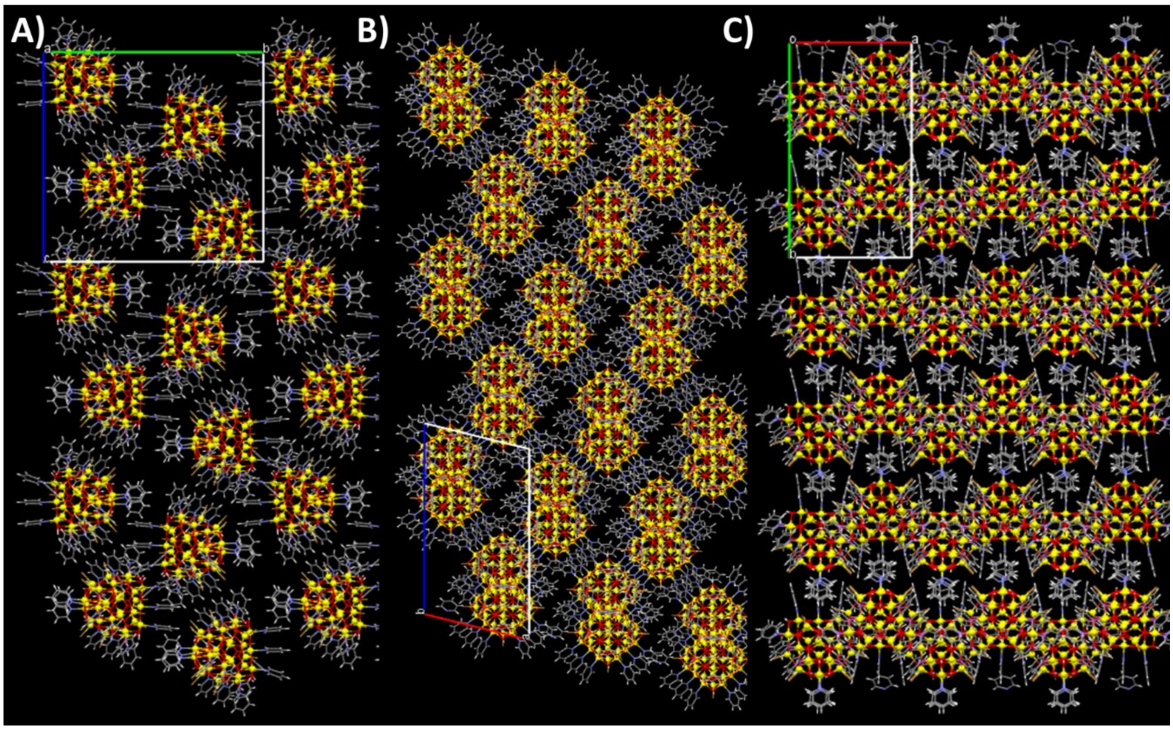

Figure S5. Crystal packing diagram showing the extended structure in **1** in ball-and-stick representation viewed down the *a*-, *b*-, and *c*-axes of the unit cell (left-right, respectively). Br counter anions omitted.

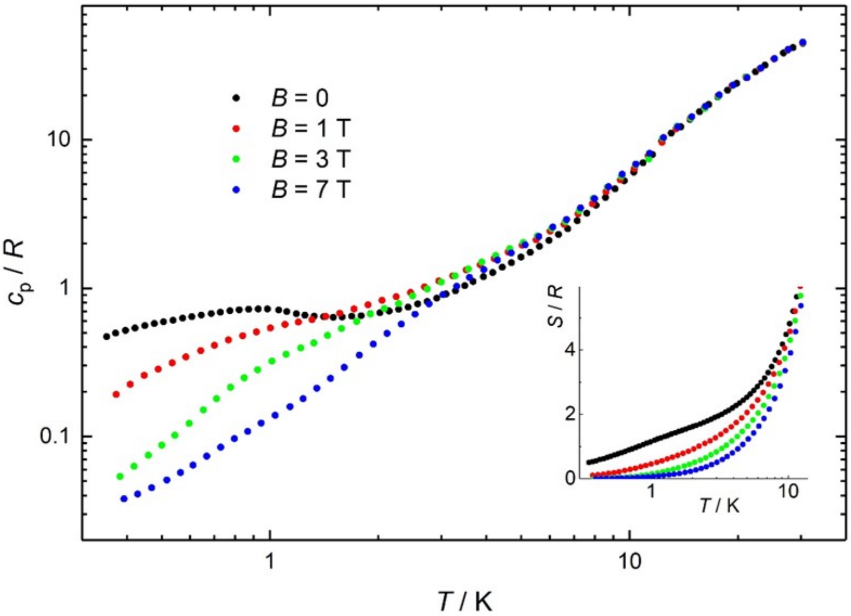

Figure S6: Temperature dependence of the low-temperature experimental heat capacity, c_p/R, and entropy, $S/R = \int c_p/R\mathrm{d}T$ (inset), normalised to the gas constant, *R*, for selected values of the applied magnetic field, *B*, as labelled. In agreement with the susceptibility data (Fig. 4), both sets of data become field-dependent on lowering the temperature below ~10 K, weakly at first and then stronger for $T < 2$ K. In further agreement with the magnetic data, the heat capacity and entropy are very small at the lowest temperatures. For instance, the zero-field magnetic entropy content reaches ca. $S = 1.6\ R$ at $T = 2$ K, which is significantly smaller than that expected for 34 uncoupled Fe^{III} spins, *i.e.*, $S = 34 \times \ln(6) = 60.9\ R$.

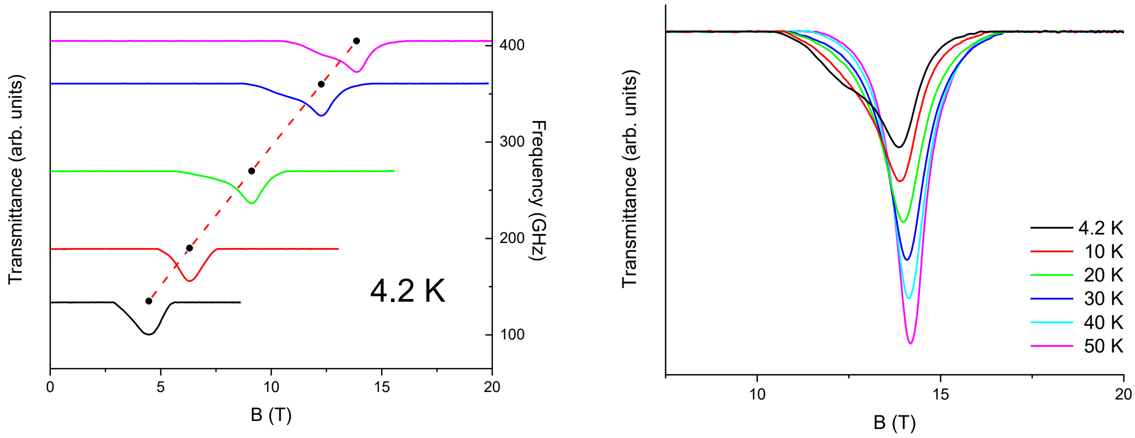

Figure S7: (left) HFEPR spectra of a polycrystalline sample of **1** recorded at 4.2 K and frequencies between 135-405 GHz (left). A linear fit of field-frequency plot affords a *g*-value of 2.04 ± 0.01, and extrapolation of the field-frequency plot gives a zero field resonance frequency of 8.10 GHz (0.26 cm^{-1}). (right) HFEPR spectra at 405 GHz and tempearatures between 4.2 - 50 K, revealing a narrowing and shift of the resonance field position to higher field with increasing temperature. The asymmetry / line broadening observed is likely due to one of, or a combination of (a) multiple transitions within multiple *S* states, (b) anisotropy, and (c) correlation effects. [4b] The Fe_{34} cluster demonstartes very strong exchange coupling between the Fe centres, and given the relative symmetry, one would expect the local anisotropies of different Fe sites to cancel out and the effective local anisotropy of the whole cluster to be small.

Computational Details

We have used the diamagnetic substitution method to calculate the exchange coupling constants in **1** employing the Gaussian 09 suite.[5] Since the calculation on the full structure of **1** is prohibitively expensive and time-consuming, we have divided the Fe_{34} cluster into five model complexes in order to calculate the five exchange coupling constants (J_1-J_5). See Figures S8-S12 below. In these models, the surrounding Fe(III) ions not involved in the pairwise exchange interaction under investigation were substituted by diamagnetic Ga(III) ions in order to maintain the same coordination environment. The exchange coupling constants were estimated using the broken symmetry approach developed by Noodleman.[6] Ahlrichs' triple-ξ plus polarisation basis set was used for the Fe, O, Br and N atoms, while the split valance plus polarisation basis set was used for Ga, C, and H atoms.[7] All theoretical calculations have been performed using the B3LYP functional since it has been proven to produce excellent estimates of *J* values.[8],[9],[10] Exchange coupling constants have been derived from the difference between the broken symmetry (BS) and high spin (HS) state, and the quardatic conversion method.

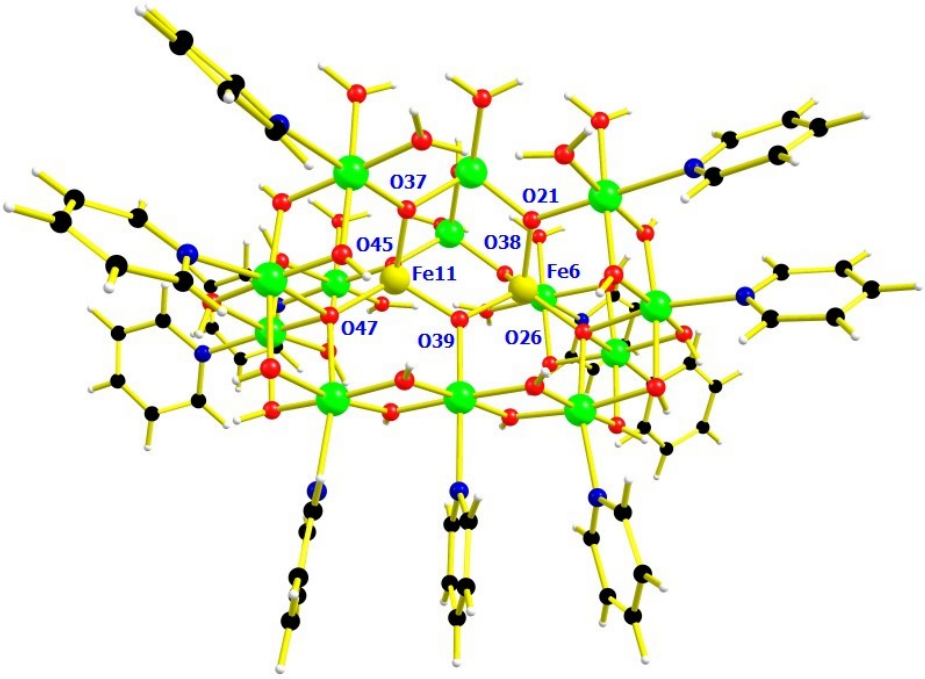

Figure S8. Model structure used to calculate the J_1 exchange. Colour Code: Fe – Yellow; Ga – Green; Br - Brown; O – Red; N – Blue; C – Black; H - White.

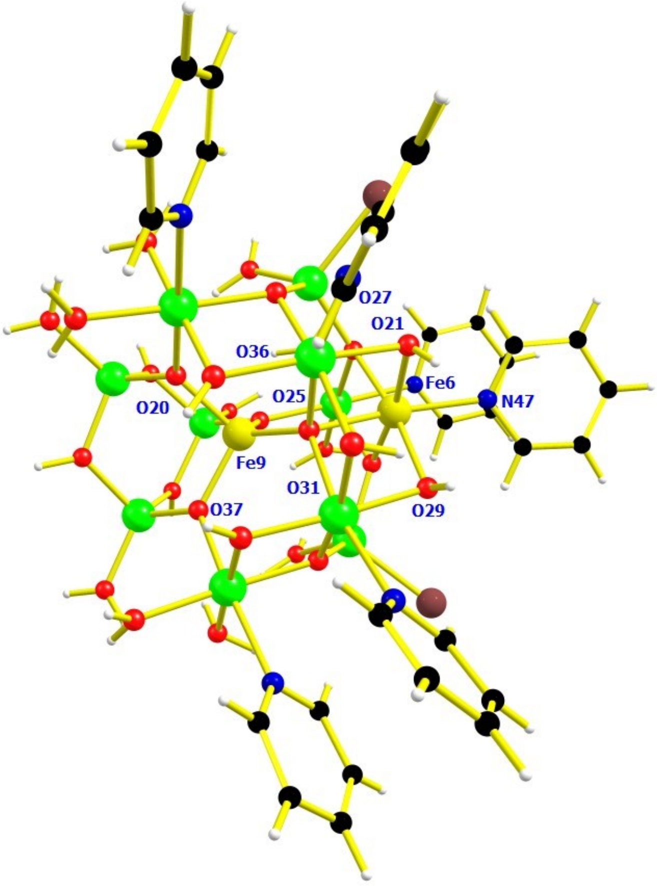

Figure S9. Model structure used to calculate the J_2 exchange. Colour Code: Fe – Yellow; Ga – Green; Br - Brown; O – Red; N – Blue; C – Black; H - White.

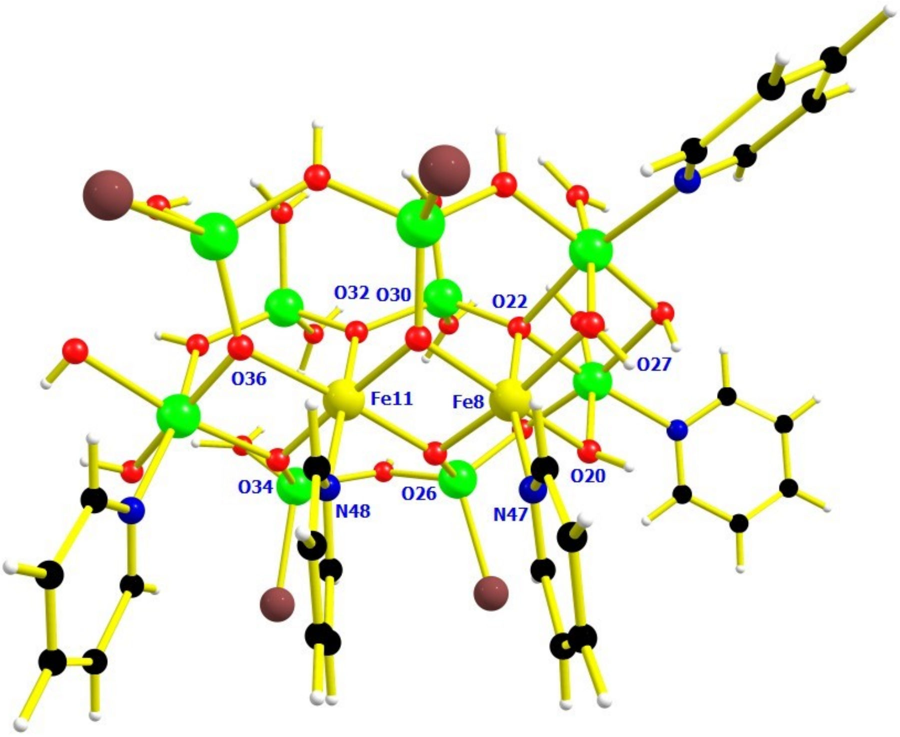

Figure S10. Model structure to calculate the J_3 exchange. Colour Code: Fe – Yellow; Ga – Green; Br - Brown; O – Red; N – Blue; C – Black; H - White.

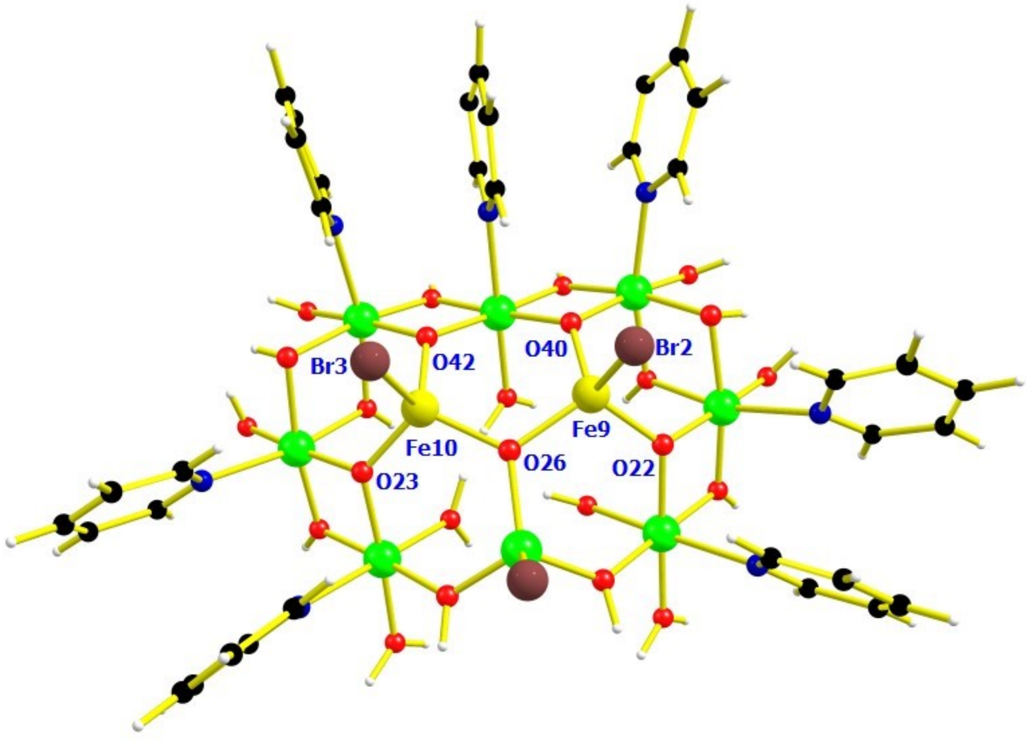

Figure S11. Model structure used to calculate the J_4 exchange. Colour Code: Fe – Yellow; Ga – Green; Br - Brown; O – Red; N – Blue; C – Black; H - White.

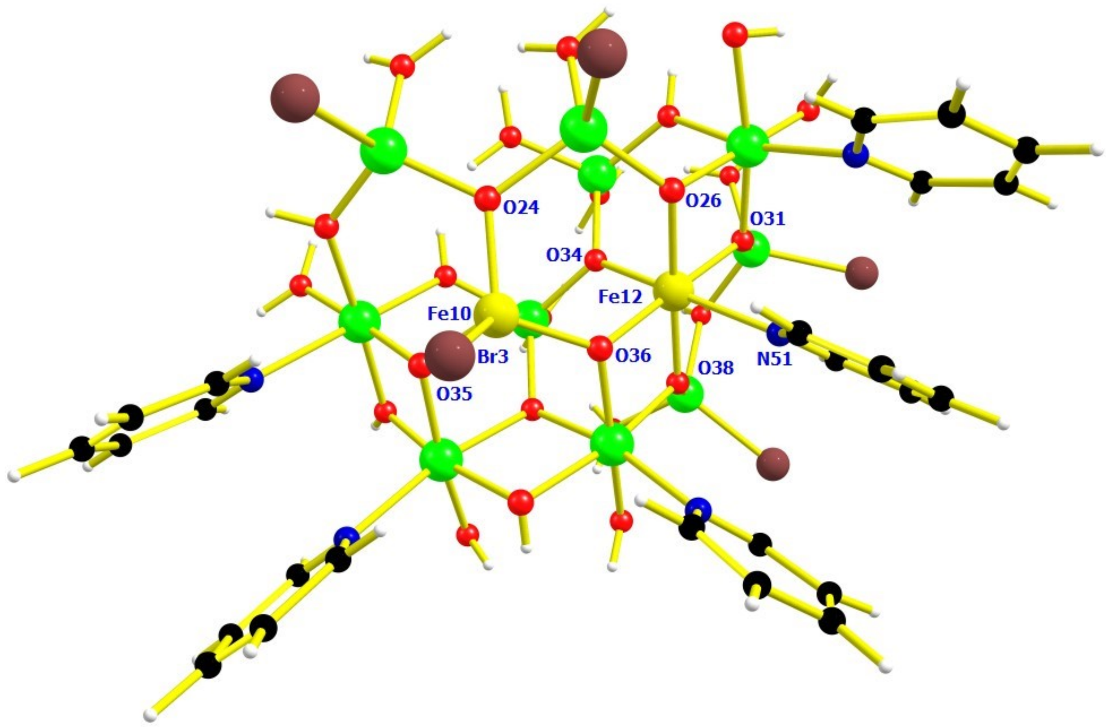

Figure S12. Model structure to calculate the J_5 exchange. Colour Code: Fe – Yellow; Ga – Green; Br - Brown; O – Red; N – Blue; C – Black; H - White.

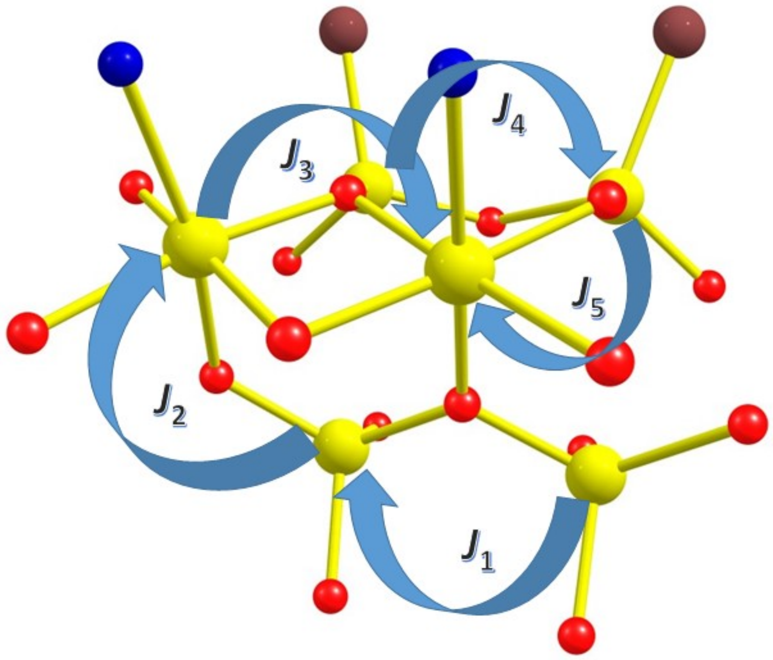

$$\widehat{H} = -6J_1 \left[\hat{S}_{Itd} - \hat{S}_{Itd}\right] - 24J_2 \left[\hat{S}_{Itd} - \hat{S}_{oct}\right] - 25J_3 \left[\hat{S}_{oct} - \hat{S}_{oct}\right] - 12J_4 \left[\hat{S}_{Otd} - \hat{S}_{Otd}\right] - 48J_5 \left[\hat{S}_{Otd} - \hat{S}_{oct}\right] \quad (1)$$

$$H = -J_1 \left[\widehat{S_1}\hat{S}_2 + \hat{S}_1\hat{S}_3 + \widehat{S_1}\hat{S}_4 + \hat{S}_2\hat{S}_4 + \hat{S}_2\hat{S}_3 + \hat{S}_3\hat{S}_4\right] - J_2\left[\hat{S}_1\hat{S}_8 + \hat{S}_1\hat{S}_{34} + \widehat{S_1}\hat{S}_6 + \widehat{S_1}\hat{S}_5 + \widehat{S_1}\hat{S}_{10} + \hat{S}_{17} + \hat{S}_2\hat{S}_{27} + \hat{S}_2\hat{S}_{28} + \hat{S}_2\hat{S}_{31} + \hat{S}_2\hat{S}_{30} + \hat{S}_2\hat{S}_5 + \hat{S}_2\hat{S}_{25} + \hat{S}_3\hat{S}_{12} + \hat{S}_3\hat{S}_{13} + \hat{S}_3\hat{S}_{14} + \hat{S}_3\hat{S}_{10} + \hat{S}_3 S_{25} + \hat{S}_3\hat{S}_{22} + \hat{S}_4\hat{S}_{19} + \hat{S}_4\hat{S}_{20} + \hat{S}_4\hat{S}_{21} + S_4\hat{S}_{17} + \hat{S}_4\hat{S}_{30} + \hat{S}_4\hat{S}_{22}\right] - J_3\left[\hat{S}_{31}\hat{S}_{28} + \hat{S}_{28}\hat{S}_{30} + \hat{S}_{30}\hat{S}_{20} + \hat{S}_{20}\hat{S}_{21} + \hat{S}_{21}\hat{S}_{22} + \hat{S}_{22}\hat{S}_{13} + \hat{S}_{13}\hat{S}_{14} + \hat{S}_{13}\hat{S}_{12} + \hat{S}_{14}\hat{S}_{12} + \hat{S}_{14}\hat{S}_{10} + \hat{S}_{10}\hat{S}_8 + \hat{S}_8\hat{S}_6 + \hat{S}_8\hat{S}_{34} + \hat{S}_6\hat{S}_5 + \hat{S}_5\hat{S}_{31} + \hat{S}_{34}\hat{S}_9 + \hat{S}_{31}\hat{S}_{27} + + \hat{S}_{34}\hat{S}_{17} + \hat{S}_{28}\hat{S}_{27} + \hat{S}_{27}\hat{S}_{25} + \hat{S}_{25}\hat{S}_{12} + \hat{S}_{19}\hat{S}_{17} + \hat{S}_{19}\hat{S}_{20} + \widehat{S_{19}}\hat{S}_{21} + \hat{S}_6\hat{S}_{34}\right] - J_4\left[\hat{S}_7\hat{S}_{11} + \hat{S}_7\hat{S}_{26} + \hat{S}_{11}\hat{S}_{26} + \hat{S}_{18}\hat{S}_{33} + \hat{S}_{18}\hat{S}_{32} + \hat{S}_{32}\hat{S}_{33} + \hat{S}_9\hat{S}_{16} + \hat{S}_9\hat{S}_{15} + \hat{S}_{15}\hat{S}_{16} + \hat{S}_{23}\hat{S}_{24} + \hat{S}_{23}\hat{S}_{29} + S_{24}\hat{S}_{29}\right] - J_5\left[\widehat{S_9}\hat{S}_{17} + \widehat{S_9}\hat{S}_{34} + \hat{S}_9\hat{S}_8 + \hat{S}_9\hat{S}_{10} + \hat{S}_{16}\hat{S}_{17} + \hat{S}_{16}\hat{S}_{19} + \hat{S}_{16}\hat{S}_{21} + \hat{S}_{16}\hat{S}_{22} + \hat{S}_{15}\hat{S}_{10} + \hat{S}_{15}\hat{S}_{14} + \hat{S}_{15}\hat{S}_{22} + \hat{S}_{15}\hat{S}_{13} + \hat{S}_7\hat{S}_8 + \hat{S}_7\hat{S}_{10} + \hat{S}_7\hat{S}_6 + \hat{S}_7\hat{S}_5 + \hat{S}_{11}\hat{S}_{12} + \hat{S}_{11}\hat{S}_{25} + \hat{S}_{11}\hat{S}_{14} + \hat{S}_{11}\hat{S}_{10} + \hat{S}_{26}\hat{S}_{25} + \hat{S}_{26}\hat{S}_{27} + \hat{S}_{26}\hat{S}_5 + \hat{S}_{26}\hat{S}_{31} + \hat{S}_{32}\hat{S}_{28} + \hat{S}_{32}\hat{S}_{30} + \hat{S}_{32}\hat{S}_5 + \hat{S}_{32}\hat{S}_{31} + \hat{S}_{18}\hat{S}_{19} + \hat{S}_{18}\hat{S}_{20} + \hat{S}_{18}\hat{S}_{30} + \hat{S}_{18}\hat{S}_{17} + \hat{S}_{23}\hat{S}_{22} + \hat{S}_{23}\hat{S}_{13} + \hat{S}_{23}\hat{S}_{25} + \hat{S}_{23}\hat{S}_{12} + \hat{S}_{29}\hat{S}_{27} + \hat{S}_{29}\hat{S}_{28} + \hat{S}_{29}\hat{S}_{30} + \hat{S}_{29}\hat{S}_{25} + \hat{S}_{24}\hat{S}_{20} + \hat{S}_{24}\hat{S}_{30} + \hat{S}_{24}\hat{S}_{21} + \hat{S}_{24}\hat{S}_{22} + \hat{S}_{33}\hat{S}_{17} + \hat{S}_{33}\hat{S}_{34} + \hat{S}_{33}\hat{S}_6 + \hat{S}_{33}\hat{S}_5\right] \quad (2)$$

Scheme S1. Schematic of the five different exchange interactions present in **1**, together with the exchange part of the corresponding spin-Hamiltonian (1), and the expanded total exchange spin Hamiltonian (2). $\hat{S}_{Itd}$ is the spin operator of the inner tetrahedral ions, $\hat{S}_{oct}$ the octahedral ions, and $\hat{S}_{Otd}$ the outer tetrahedral ions.

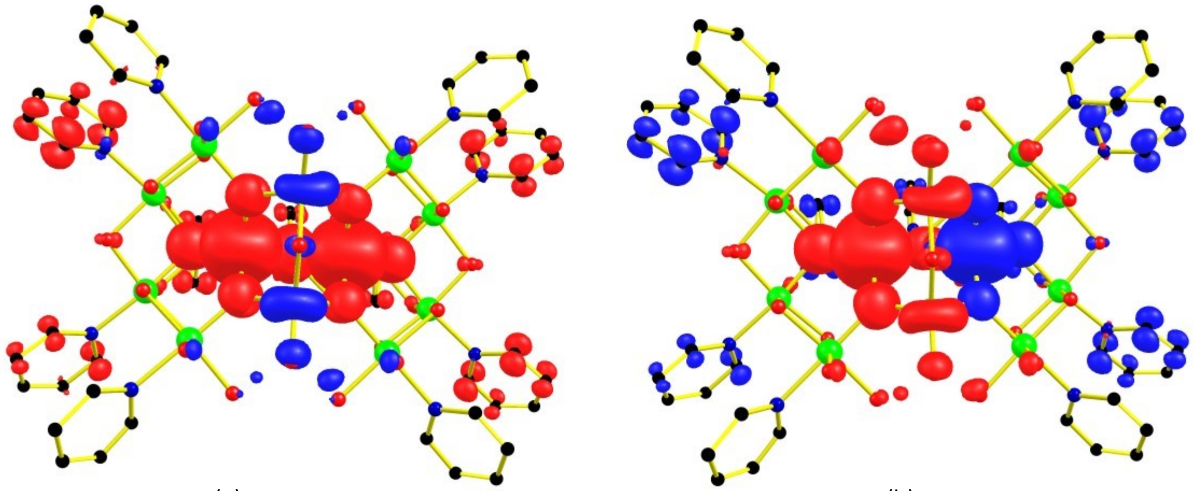

(a) (b)

Figure S13: Spin density plots for the J_1 model complex exchange pathway; (a) High Spin, (b) Broken Symmetry. The isodensity surface shown corresponds to a value of 0.003 e -/bohr^3. The red and blue surfaces represent positive and negative spin density, respectively. Colour Code: Fe – Yellow; Ga – Green; Br - Brown; O – Red; N – Blue; C – Black. Hydrogen atoms are omitted for clarity.

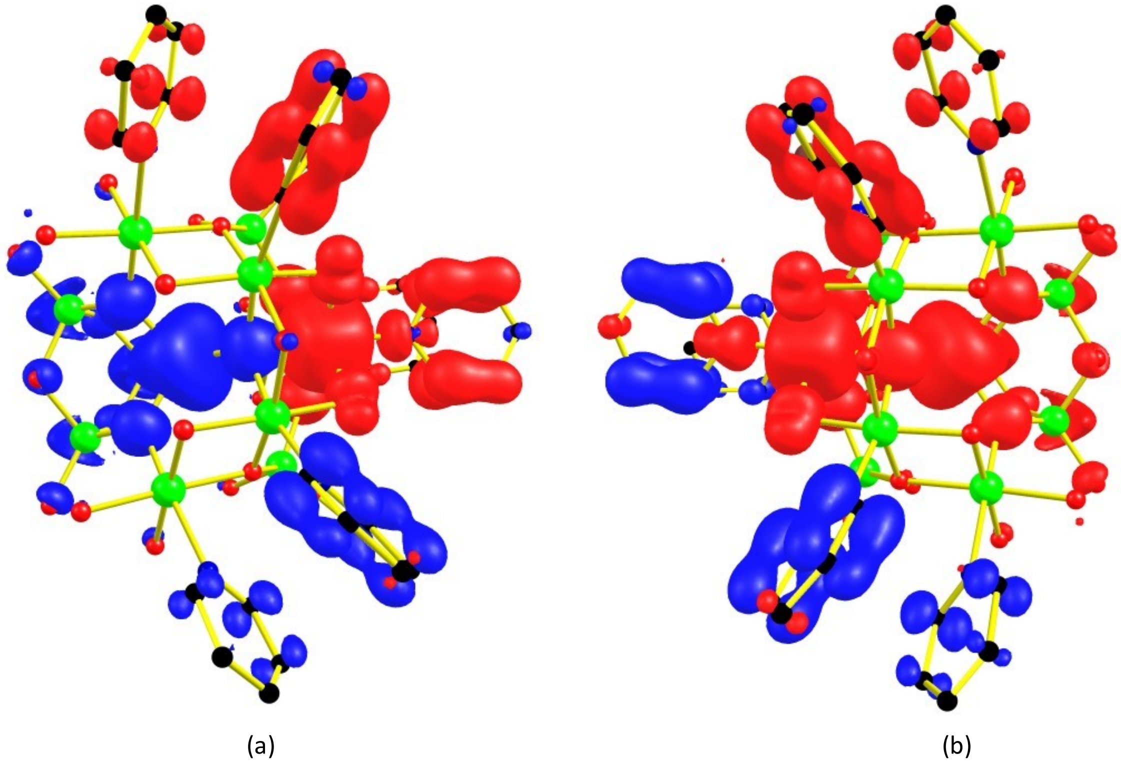

(a) (b)

Figure S14: Spin density plots for the J_2 model complex exchange pathway; (a) High Spin, (b) Broken Symmetry. The isodensity surface shown corresponds to a value of 0.003 e -/bohr^3. The red and blue surfaces represent positive and negative spin density, respectively. Colour Code: Fe – Yellow; Ga – Green; Br - Brown; O – Red; N – Blue; C – Black. Hydrogen atomss are omitted for clarity.

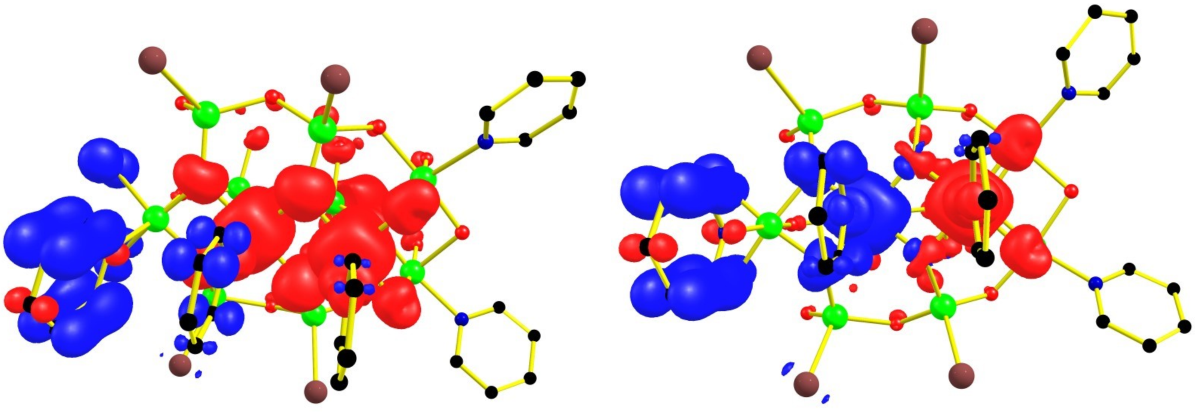

(a) (b)

Figure S15: Spin density plots for the J_3 model complex exchange pathway; (a) High Spin, (b) Broken Symmetry. The isodensity surface shown corresponds to a value of 0.003 e -/bohr^3. The red and blue surfaces represent positive and negative spin density, respectively. Colour Code: Fe – Yellow; Ga – Green; Br - Brown; O – Red; N – Blue; C – Black. Hydrogen atoms are omitted for clarity.

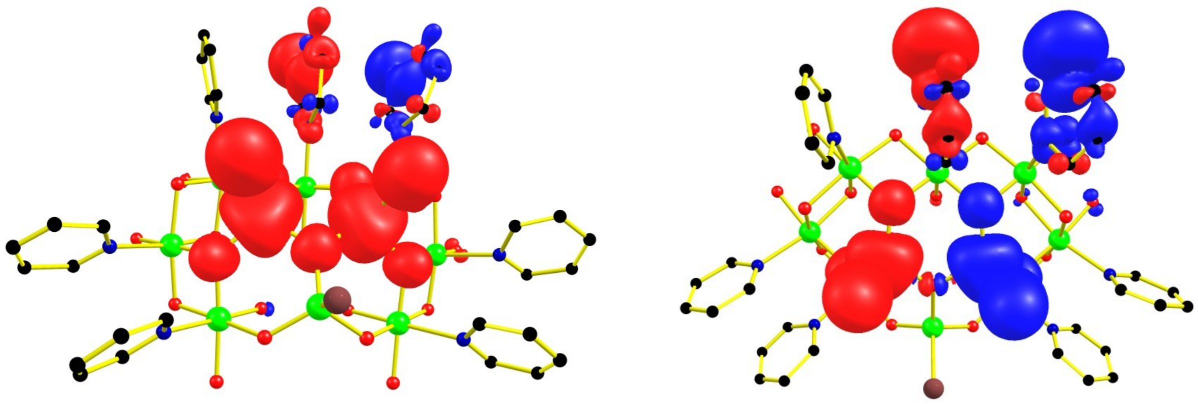

(a) (b)

Figure S16: Spin density plots for the J_4 model complex exchange pathway; (a) High Spin, (b) Broken Symmetry. The isodensity surface shown corresponds to a value of 0.003 e -/bohr^3. The red and blue surfaces represent positive and negative spin density, respectively. Colour Code: Fe – Yellow; Ga – Green; Br - Brown; O – Red; N – Blue; C – Black. Hydrogen atoms are omitted for clarity.

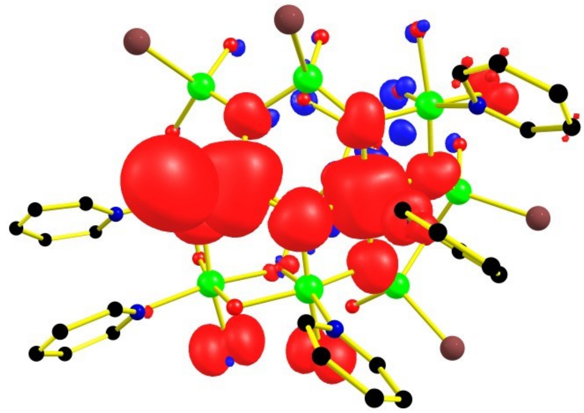

(a)

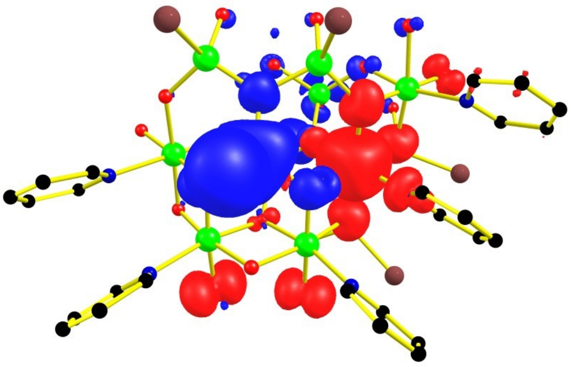

(b)

Figure S17: Spin density plots for the J_5 model complex exchange pathway; (a) High Spin, (b) Broken Symmetry. The isodensity surface shown corresponds to a value of 0.062 e -/bohr^3. The red and blue surfaces represent positive and negative spin density, respectively. Colour Code: Fe – Yellow; Ga – Green; Br - Brown; O – Red; N – Blue; C – Black. Hydrogens are omitted for clarity.

Table S2. The Mulliken spin density of the atoms surrounding the Fe(III) centre in the J_1 exchange pathway (see the atom numbering in Figure S8).

Atom	HS	BS
**Fe6**	4.077	-4.015
**Fe11**	4.090	4.134
**O39**	0.406	0.085
**O37**	0.121	0.127
**O47**	0.206	0.227
**O45**	0.122	0.130
**O26**	0.202	-0.183
**O21**	0.118	-0.108
**O38**	0.115	-0.106

Table S3. The Mulliken spin density of the atoms surrounding the Fe(III) centre in the J_2 exchange pathway (see the atom numbering in Figure S9).

Atom	HS	BS
**Fe6**	4.286	4.266
**Fe9**	4.006	-4.002
**O25**	0.286	-0.156
**O29**	0.107	0.091
**O27**	0.136	0.127
**O21**	0.124	0.110
**O31**	0.129	0.119
**N47**	0.146	0.031
**O36**	0.104	-0.102
**O37**	0.108	-0.108
**O20**	0.106	-0.106

Table S4. The Mulliken spin density of the atoms surrounding the Fe(III) centre in the J_3 exchange pathway (see the atom numbering in Figure S10).

Atom	HS	BS
**Fe8**	4.252	4.250
**Fe11**	4.253	-4.256
**O26**	0.273	0.029
**O30**	0.263	0.018
**O20**	0.111	0.108
**O27**	0.132	0.130
**O22**	0.058	0.055
**N47**	0.111	0.110
**O34**	0.132	-0.124
**O36**	0.132	-0.122
**N48**	0.109	-0.069
**O32**	0.054	-0.015

Table S5. The Mulliken spin density of the atoms surrounding the Fe(III) centre in the J_4 exchange pathway (see the atom numbering in Figure S11).

Atom	HS	BS
**Fe9**	4.095	-4.074
**Fe10**	4.088	4.072
**O26**	0.202	0.001
**O23**	0.187	0.186
**O42**	0.243	0.242
**Br3**	0.312	0.307
**O40**	0.236	-0.232
**O22**	0.181	-0.178
**Br2**	0.321	-0.315

Table S6. The Mulliken spin density of the atoms surrounding the Fe(III) centre in the J_5 exchange pathway (see the atom numbering in Figure S12).

Atom	HS	BS
**Fe10**	4.081	-4.051
**Fe12**	4.259	4.222
**O36**	0.365	-0.082
**O38**	0.122	0.117
**O31**	0.111	0.109
**O26**	0.119	0.115
**O34**	0.016	0.013
**N51**	0.091	0.090
**O35**	0.184	-0.185
**O24**	0.076	--0.079
**Br3**	0.351	-0.343

Table S7. Overlap integral between the 3d orbitals of the Fe(III) ions in the J_1 exchange pathway. The most significant are highlighted in yellow.

Beta→ Alpha	d_{z2}	d_{xy}	d_{xz}	$d_{x^2-y^2}$	d_{yz}
d_{yz}	0.175	-0.027	0.175	-0.000	-0.234
d_{xy}	0.021	0.041	0.076	0.041	-0.082
d_{z2}	-0.073	0.013	-0.144	-0.093	-0.216
$d_{x^2-y^2}$	-0.066	0.063	-0.024	-0.331	0.058
d_{xz}	-0.262	0.003	-0.143	0.153	-0.058

Table S8. Overlap integral between the 3d orbitals of the Fe(III) ions in the J_2 exchange pathway. The most significant are highlighted in yellow.

Beta→ Alpha	d_{yz}	$d_{x^2-y^2}$	d_{xy}	d_{xz}	d_{z2}
$d_{x^2-y^2}$	0.141	-0.007	0.184	-0.236	-0.098
d_{yz}	-0.033	0.319	0.081	-0.202	-0.151
d_{xz}	0.123	0.196	0.062	0.036	-0.270
d_{xy}	0.117	-0.089	-0.103	0.243	0.282
d_{z2}	-0.013	0.071	0.035	-0.113	0.099

Table S9. Overlap integral between the 3d orbitals of the Fe(III) ions in the J_3 exchange pathway. The most significant are highlighted in yellow.

Beta→ Alpha↓	d_{xz}	d_{xy}	d_{yz}	$d_{x^2-y^2}$	d_{z2}
d_{xz}	0.021	0.011	-0.003	0.014	-0.044
d_{xy}	-0.041	0.197	-0.047	-0.036	-0.121
d_{yz}	-0.110	-0.002	-0.158	0.045	-0.178
$d_{x^2-y^2}$	-0.027	-0.317	-0.081	-0.171	-0.068
d_{z2}	-0.106	0.047	0.031	-0.012	-0.017

Table S10. Overlap integral between the 3d orbitals of the Fe(III) ions in the J_4 exchange pathway. The most significant are highlighted in yellow.

Beta→ Alpha	d_{z2}	d_{xz}	d_{yz}	d_{xy}	$d_{x^2-y^2}$
d_{z2}	0.264	0.266	-0.037	0.032	0.103
d_{xy}	0.077	0.053	0.024	0.018	-0.008
d_{xz}	-0.068	0.119	0.119	-0.285	-0.177
$d_{x^2-y^2}$	0.014	0.000	0.364	-0.113	0.001
d_{yz}	-0.012	-0.038	-0.087	0.019	-0.213

Table S11. Overlap integral between the 3d orbitals of the Fe(III) ions in the J_5 exchange pathway. The most significant are highlighted in yellow.

Beta→ Alpha↓	d_{x2-y2}	d_{yz}	d_{xz}	d_{xy}	d_{z^2}
d_{z2}	0.106	0.220	0.262	0.025	-0.011
d_{xy}	0.071	-0.007	-0.011	-0.148	-0.005
$d_{x^2-y^2}$	-0.136	0.223	0.238	0.013	-0.002
d_{yz}	-0.002	0.054	0.212	-0.073	0.027
d_{xz}	-0.005	0.117	0.278	-0.055	-0.264

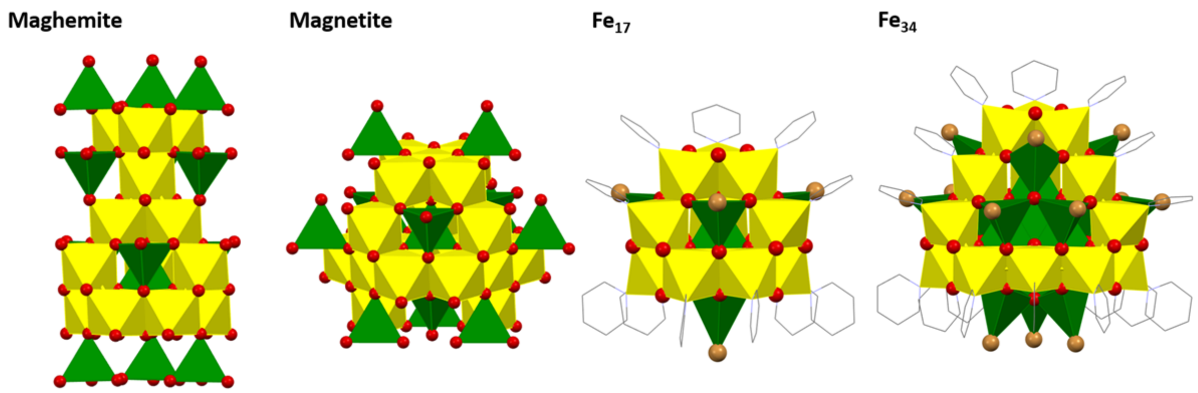

Figure S18: A structural comparison between the minerals maghemite (left) and magnetite (2nd left) with [Fe_{17}] and [Fe_{34}] (right), highlighting the positions of the tetrahedral (green) and octahedral (yellow) Fe(III) ions.

References

[1] G. M. Sheldrick, *Acta Crystallogr. Sect. C: Cryst. Struct. Commun.* **2015**, *71*, 3-8.

[2] O. V. Dolomanov, L. J. Bourhis, R. J. Gildea, J. A. K. Howard, H. Puschmann, *J. Appl. Crystallogr.* **2009**, *42*, 339-341.

[3] H. Nojiri, K.-Y. Choi, N. Kitamura, *J. Magn. Mater.*, **2007**, *310*, 1468-1472.

[4] a) H. Nojiri, Y. Ajiro, T. Asano, J.-P. Boucher, *New J. Phys.*, **2006**, *8*, 218; b) O. Cépas, T. Ziman, *Progress of Theoretical Physics Supplement*, 2005, 159, 280–291.

[5] M. J. Frisch, G. W. Trucks, H. B. Schlegel, G. E. Scuseria, M. A. Robb, J. R. Cheeseman, G. Scalmani, V. Barone, G. A. Petersson, H. Nakatsuji, X. Li, M. Caricato, A. Marenich, J. Bloino, B. G. Janesko, R. Gomperts, B. Mennucci, H. P. Hratchian, J. V. Ortiz, A. F. Izmaylov, J. L. Sonnenberg, D. Williams-Young, F. Ding, F. Lipparini, F. Egidi, J. Goings, B. Peng, A. Petrone, T. Henderson, D. Ranasinghe, V. G. Zakrzewski, J. Gao, N. Rega, G. Zheng, W. Liang, M. Hada, M. Ehara, K. Toyota, R. Fukuda, J. Hasegawa, M. Ishida, T. Nakajima, Y. Honda, O. Kitao, H. Nakai, T. Vreven, K. Throssell, J. A. Montgomery Jr., J. E. Peralta, F. Ogliaro, M. Bearpark, J. J. Heyd, E. Brothers, K. N. Kudin, V. N. Staroverov, T. Keith, R. Kobayashi, J. Normand, K. Raghavachari, A. Rendell, J. C. Burant, S. S. Iyengar, J. Tomasi, M. Cossi, J. M. Millam, M. Klene, C. Adamo, R. Cammi, J. W. Ochterski, R. L. Martin, K. Morokuma, O. Farkas, J. B. Foresman, D. J. Fox, Gaussian 09, Revision A.02, Gaussian Inc., Wallingford CT, **2016**.

[6] L. Noodleman, *J. Chem. Phys*. **1981**, *74*, 5737–5743.

[7] A. Schäfer, C. Huber, R. Ahlrichs, *J. Chem. Phys*. **1994**, *100*, 5829.

[8] D. Becke, *J. Chem. Phys*. **1993**, *98*, 5648–5652.

[9] A. Schäfer, H. Horn, R. Ahlrichs, *J. Chem. Phys*. **1992**, *97*, 2571–2577.

[10] A. Schäfer, C. Huber and R. Ahlrichs, *J. Chem. Phys*. **1994**, *100*, 5829–5835.
